# Carcinoma Ex-Pleomorphic Adenoma of the Lacrimal Gland with Intracranial Extension

**DOI:** 10.1055/a-2629-9251

**Published:** 2025-06-23

**Authors:** Daniel Sharbel, Osayamen Atekha, Scott Rahimi, James Kenneth Byrd

**Affiliations:** 1 Department of Otolaryngology-Head and Neck Surgery, Medical College of Georgia at Augusta University, Augusta, Georgia, United States; 2 Medical College of Georgia at Augusta University, Augusta, Georgia, United States; 3 Department of Neurological Surgery, Medical College of Georgia at Augusta University, Augusta, Georgia, United States

**Keywords:** carcinoma ex-pleomorphic adenoma, lacrimal gland, skull base, free flap

## Abstract

Carcinoma ex-pleomorphic adenoma (CXPA) of the lacrimal gland is rare, and its management can be complex given its anatomic location. In this case report, we describe our approach to multidisciplinary management of a CXPA with intracranial extension.

## Case Description

**Fig. 1:**
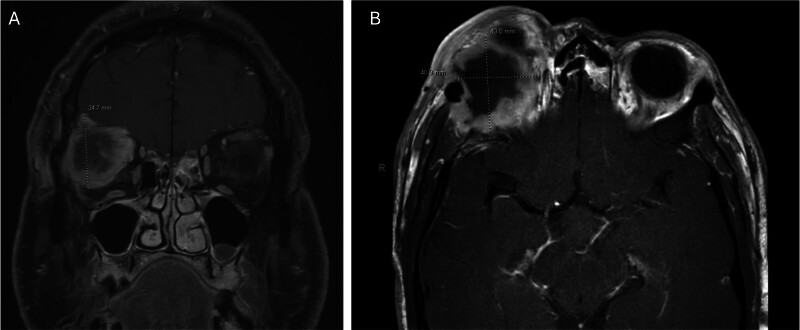
Coronal ( **A** ) and axial ( **B** ) T1-weighted magnetic resonance imaging demonstrating an enhancing, destructive orbital mass with intracranial extent.

A 47-year-old man with neurofibromatosis type I exhibited a rapidly enlarging right orbital mass, deteriorating vision, and facial pain. Magnetic resonance imaging (MRI) identified a 4.0 × 4.1 × 3.5 cm enhancing orbital mass with diffusion restriction, bone erosion, and intracranial involvement ( Fig. 1A, B ). There was mild pachymeningeal enhancement and minimal T2 FLAIR signal hyperintensity in the right frontal lobe. Malignant cytological features without further histological definition were confirmed via fine-needle aspiration.

## Intervention

Multidisciplinary tumor board discussion led to recommendation of orbital exenteration, orbitocranial craniotomy, and resection of intracranial disease followed by adjuvant therapy as indicated by final pathology. Reconstruction involved defect coverage with a pericranial flap, remnant craniotomy bone flap, synthetic dural matrix and sealant, and left anterolateral thigh free flap. Initial free flap reconstruction was complicated by multiple arterial thromboses and immediate flap failure. The decision was then made to perform a second-stage flap reconstruction with utilization of low-intensity heparin infusion. Following initial surgery, computed tomography imaging revealed a small intraparenchymal hemorrhage within the right middle/inferior frontal gyri with mild surrounding edema without significant mass effect. A subsequent successful latissimus dorsi free flap reconstruction was performed 48 hours after an initial surgery.

## Outcomes

**Fig. 2:**
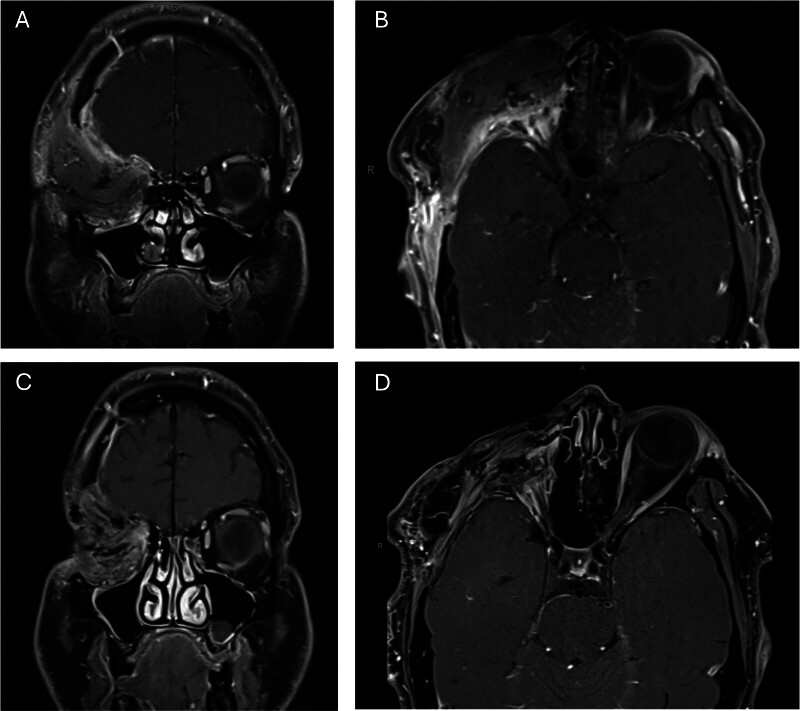
( **A, B** ) Two-month postoperative coronal ( **A** ) and axial ( **B** ) T1-weighted magnetic resonance imaging (MRI) showing postsurgical changes with generalized soft tissue enhancement and no evidence of local recurrence or residual tumor. ( **C, D** ) One-year postoperative coronal ( **C** ) and axial ( **D** ) T1-weighted MRI showing residual dural thickening and postsurgical changes without evidence of local recurrence.

Postoperative imaging showed no intracranial hemorrhage progression, and the patient was discharged after an uneventful hospital course on postoperative day 8. Pathology reported high-grade adenocarcinoma with PLAG1–RP1 fusion, indicative of carcinoma ex-pleomorphic adenoma (CXPA). Follow-up MRI at 2 months after surgery and 1 year indicated no recurrence ( Fig. 2A–D ).

## Discussion

This case highlights the locally aggressive nature of lacrimal CXPA, and the surgical complexities involved in management of lacrimal malignancy. Lacrimal tumors represent 10% of all space-occupying lesions of the orbit. 1 CXPA is the second most common lacrimal gland malignancy, but its overall incidence is relatively low, comprising 8% of all lacrimal gland epithelial tumors. Histologically, PLAG–RP1 fusion is a useful specific marker for identifying CXPA, especially those of salivary gland origin. 2 3 Prognosis is relatively favorable with 5-year overall and cancer-specific survival of 81.9%. 4 Signs and symptoms at presentation typically include rapid eyelid swelling, proptosis, pain, and vision change. 5 Treatment strategies involve surgical resection and adjuvant therapy as indicated by pathologic features. A globe-sparing approach is reasonable in early-stage tumors, but this requires multidisciplinary evaluation and careful consideration of patient-specific factors. More advanced cases typically require orbital exenteration. 6 Patients who require free tissue transfer may be at significantly increased risk of flap vessel thrombosis if their defect involves dura and/or brain parenchyma. 7 Despite the potential risks, aggressive surgical management with a multidisciplinary approach can lead to favorable outcomes in cases of advanced tumors.

## Conclusion

This case underscores the importance of a multidisciplinary approach to managing lacrimal malignancies, particularly those with intracranial extent. Aggressive surgical management is required for definitive treatment of advanced local disease. Careful reconstructive planning is necessary for patients with composite skull base defects.
